# Case Illustrating the Effect of Local Irritation, in Exciting and Maintaining a State of Great Constitutional Derangement

**Published:** 1826-11

**Authors:** H. Earle

**Affiliations:** St. Bartholomew's Hospital.


### LOCAL IRRITATION.

*Case illustrating the Effect of Local Irritation, in exciting and maintaining a State of great Constitutional Derangement.* By H. Earle, F.R.S. &c. (St. Bartholomew's Hospital.)

William Ferrein, aged twenty-three, a fine muscular young man, was admitted into Powel's Ward, at St. Bartholomew's Hospital, February 2d, 1826, on account of a disease of the thigh-bone, the consequence of a gun-shot wound. It appeared that his thigh had been broken by a musket shot, at Sencamporne in the East Indies, December 17th, 1822. The limb was set the following day, and the ball removed; and he went on well for six weeks, when, from some carelessness, the fracture was displaced. He went on shore at Calcutta; the bone was reset, and he continued slowly improving for six months, when abscess formed. Since that time there have been, at short intervals, repeated abscesses, accompanied with much constitutional disturbance. About the centre of the front of the thigh, where the ball entered, there is a large cicatrix, in the middle of which there was a fistulous opening. The thigh-bone at this part appeared much enlarged, forming an extensive bony case. A probe, passed into the fistulous opening, led into the centre of this bony case, but did not detect any portion of dead bone. The thigh had been extensively laid open at this part, without detecting any sequestra, or affording any relief.

After his admission into the hospital, issues were made in the neighbourhood of the supposed diseased bone, and his health strictly attended to. Under this treatment the opening closed, but repeatedly opened again, and gave vent to a thin semi-transparent discharge, almost resembling sinovia. During the formation of these abscesses, he suffered severely in his health, and had many severe rigors. He appeared, indeed, constantly suffering from the irritation of the affected limb, and greatly disposed to inflammatory attacks.

About the middle of May, he had a severe attack of acute rheumatism affecting all his joints, which receded, and fell upon his chest and diaphragm, requiring the most active treatment, with copious bleeding and colchicum. He suffered two relapses; and subsequently became subject to well-marked intermittent, which was relieved by bark and ammonia.

Worn out with such repeated severe attacks, he earnestly solicited to have his limb amputated. As the joints were still quite perfect, and the limb not much wasted, I was very unwilling to accede to his wishes: more especially as the enlargement of the femur extended nearly to the trochanter, and it would have been necessary to have amputated just below the joint.

Conceiving that there might be some portion of cloth, or other foreign body, keeping up irritation, I determined to make very free incisions in the limb, before I resorted to so severe a remedy as amputation. In examining the limb with much attention, I felt a very obscure, slightly elastic sensation, at the outer and lower part of the thigh. There was no swelling perceptible, but, on pressing firmly, it caused pain; and he said that he had often felt pain in that situation before, previously to the opening of the fistulous abscesses. This determined me to make a free and deep incision through the integuments and muscles: in doing so, I gave exit to a small quantity of a secretion, similar to that which flowed from the upper opening; and, on introducing my finger, I detected a large portion of dead bone quite loose. This was readily extracted, and subsequently two smaller portions. These several pieces of dead bone lay imbedded in a cavity surrounded by soft villous granulations, which cavity communicated by a small opening with the case of new bone above described. The larger portion consisted of the whole thickness of the walls of the femur, and about half its circumference, and was about two inches and a half long. It was quite bleached and inodorous, and did not bear the least appearance of having been touched by the absorbents. Excepting that it was beautifully white, it did not appear to have undergone any change from the moment it was splintered from the shaft of the bone. One of the smaller portions was a little grooved by the action of the absorbents. The wound was dressed with a little wet lint, and subsequently with bread-and-water poultice; under which it filled up with healthy granulations, and soon healed.

From the very day after the operation, all constitutional disturbance ceased; he rapidly regained his health and spirits, and was discharged quite well on the 11th of July, the operation having been performed on the 8th of June.

It rarely happens that we have more striking examples of the effect of mechanical local irritation on the constitution; nor do we often meet with cases in which the connexion between cause and effect are so unequivocally established, as in the one which has just been related.

*28, George-street; October 10th,* 1826.

